# Prediction and Analysis of the Protein Interactome in *Pseudomonas aeruginosa* to Enable Network-Based Drug Target Selection

**DOI:** 10.1371/journal.pone.0041202

**Published:** 2012-07-24

**Authors:** Minlu Zhang, Shengchang Su, Raj K. Bhatnagar, Daniel J. Hassett, Long J. Lu

**Affiliations:** 1 Division of Biomedical Informatics, Cincinnati Children’s Hospital Research Foundation, Cincinnati, Ohio, United States of America; 2 School of Computing Sciences and Informatics, University of Cincinnati, Cincinnati, Ohio, United States of America; 3 Department of Molecular Genetics, Biochemistry and Microbiology, College of Medicine, University of Cincinnati, Cincinnati, Ohio, United States of America; 4 School of Electronic and Computer Systems, University of Cincinnati, Cincinnati, Ohio, United States of America; 5 Department of Environmental Health, University of Cincinnati, Cincinnati, Ohio, United States of America; The Scripps Research Institute, United States of America

## Abstract

*Pseudomonas aeruginosa* (*PA*) is a ubiquitous opportunistic pathogen that is capable of causing highly problematic, chronic infections in cystic fibrosis and chronic obstructive pulmonary disease patients. With the increased prevalence of multi-drug resistant *PA*, the conventional “one gene, one drug, one disease” paradigm is losing effectiveness. Network pharmacology, on the other hand, may hold the promise of discovering new drug targets to treat a variety of *PA* infections. However, given the urgent need for novel drug target discovery, a *PA* protein-protein interaction (PPI) network of high accuracy and coverage, has not yet been constructed. In this study, we predicted a genome-scale PPI network of *PA* by integrating various genomic features of *PA* proteins/genes by a machine learning-based approach. A total of 54,107 interactions covering 4,181 proteins in *PA* were predicted. A high-confidence network combining predicted high-confidence interactions, a reference set and verified interactions that consist of 3,343 proteins and 19,416 potential interactions was further assembled and analyzed. The predicted interactome network from this study is the first large-scale PPI network in *PA* with significant coverage and high accuracy. Subsequent analysis, including validations based on existing small-scale PPI data and the network structure comparison with other model organisms, shows the validity of the predicted PPI network. Potential drug targets were identified and prioritized based on their essentiality and topological importance in the high-confidence network. Host-pathogen protein interactions between human and *PA* were further extracted and analyzed. In addition, case studies were performed on protein interactions regarding anti-sigma factor MucA, negative periplasmic alginate regulator MucB, and the transcriptional regulator RhlR. A web server to access the predicted PPI dataset is available at http://research.cchmc.org/PPIdatabase/.

## Introduction

*Pseudomonas aeruginosa* (*PA*) is a ubiquitous opportunistic pathogen that is especially notorious for causing highly problematic, chronic infections within the lungs of cystic fibrosis (CF) and chronic obstructive pulmonary disease (COPD) patients [1]. Complications due to intractable *PA* infections eventually compromise lung function, especially in CF patients, leading to death at an average age of ∼38 [2]. *PA* possesses a remarkable capacity to resist multiple front-line antibiotics, either intrinsically or following acquisition of resistance genes. Once colonization of the lungs with *PA* occurs, eradication of the organism is nearly impossible. Making matters worse, the increasing frequency of multi-drug resistant *PA* (MDRPA) strains has rendered ineffective many existing drugs, including the most powerful anti-pseudomonal β-lactams, such as expanded-spectrum cephalosporins and carbapenems [3], and the front-line aminoglycoside, tobramycin [4]. Unfortunately, over the past decade, we have seen an alarming failure rate of drugs in late-stage clinical development [5]. Many physicians and scientists foresee a crisis if novel therapeutic options continue to be unavailable [6].

The dominant philosophy in rational drug design, i.e., the “one gene, one drug, one disease” paradigm, focuses on the individual properties of a protein; for instance, whether it is essential for survival. However, many effective drugs with robust phenotypic effects have been found to affect a group of molecular targets rather than a single protein [7]. From a modern systems biology perspective, a protein’s importance is rarely defined by its individual biochemical function(s), but also its position in the protein-protein interaction (PPI) network, i.e., its potential for interacting with other proteins [8]. As the role of the functional dysregulation of PPIs as the underlying cause of disease is increasingly understood, network pharmacology, which advocates combination therapies targeting multiple interconnected nodes in a PPI network, represents a new venue in disease treatment [9], [10]. With the potential benefits of reducing drug toxicity and expanding opportunity space for druggable targets, this new concept has become increasingly plausible [9].

Curative drugs for *PA* infections could well arise through network pharmacology. However, at this stage, little is known about the PPI networks in *PA*. Because of the resources and time it would require, a large-scale experimental survey of the PPI network in *PA* has not yet been carried out, and no PPI information is included in the major database for the organism, Pseudomonas Genome Database (http://www.pseudomonas.com/). Knowledge of PPIs in *PA* is limited to a handful of protein pairs from individual small-scale studies [11].

In the past decade, computational methods based on a variety of principles have been developed for predicting PPIs to circumvent the expensive and labor-intensive large-scale experiments [12]–[15]. In a non-model organism, such as *PA*, the most common method is to perform homology mapping to model organisms with available large-scale PPI data [16]. Such an approach has several limitations. First, its applicability often depends on the availability of well-studied PPI networks from closely related organisms. Because of limited resources, high-throughput (HTP) experiments on determining PPIs have been carried out in only a handful of model organisms, such as yeast [17]–[21] and humans [22]–[24]. Second, the high false-positive rate (FPR) associated with HTP experiments appears inevitable, even in the most thoroughly characterized model organism, *Saccharomyces cerevisiae* [25]. Therefore, predicting PPIs by performing homology mapping to a single source of HTP PPI data in a closely related organism will likely generate a large number of spurious predictions. Third, the coverage of predictions may be limited by the often low number of orthologs between different bacteria, because of the rapid divergence during bacterial evolution. For instance, *Escherichia coli* and *PA* share only a small portion of the genome as orthologs (0.205 as measured by Jaccard Index).

In this study, we present a machine learning-based integrative approach to predict a genome-scale PPI network in *PA*. We previously developed a Bayesian network approach in the yeast *S. cerevisiae* to combine various genomic features that can better predict PPIs than does each individual feature [12], [13], [26]. We also developed a logistic regression approach to combine genomic features that are parameterized for membrane proteins to produce a membrane interactome map [27]. The advantages of an integrative approach are that, on one hand, genomic features capture information beyond the similarity in nucleotide sequences, and including novel interactions beyond orthologs can increase the prediction coverage. On the other hand, the validation of predicted interactions can be performed based on multiple resources, such as available experimental data.

To predict a PPI network in *PA*, we have collected, compiled, and integrated a variety of genomic and proteomic features of *PA* and employed a random forest algorithm to combine eight features with potential high predictive power. The predicted *PA* interactome from this study is the first large-scale PPI network in *PA* with high coverage and accuracy. A confidence score is associated with each predicted PPI representing the probability of the physical interaction or the co-involvement in a protein complex, which is different from the confidence weight of the protein functional linkage data from the STRING database [28]. Subsequent analysis, including validations based on existing small-scale PPI data as well as the network structure comparison with other model organisms, shows the validity of the predicted PPI network. Potential drug targets were predicted based mainly on their topological positions and essentiality. A set of essential functional modules in the *PA* PPI network was identified, and a map of host-pathogen interactions between human and *PA* proteins was analyzed. Case studies were performed on important *PA* proteins, including the anti-sigma factors MucA, negative regulator for alginate biosynthesis MucB, and the quorum sensing regulator RhlR, with their predicted interacting partners, shedding light on their candidacy to be effective drug targets. The rationale behind studying these particular proteins is that bacterial mutants lacking MucA or RhlR are either very sensitive to slightly acidified nitrite or commit a metabolic suicide during anaerobic growth, conditions that prevail during chronic CF and COPD airway disease [29], [30].

## Results

### Constructing Reference Sets

A major challenge of applying a supervised machine learning approach to predict PPIs in *PA* is to establish a reference dataset with high accuracy and coverage. On one hand, only a limited number of experimentally verified PPIs exist in *PA*. On the other hand, *E. coli*, the most closely related model organism that has large-scale PPI datasets, shares a small portion of orthologs (1656 proteins; 0.205 as measured by Jaccard Index) with *PA*. Therefore, a reference interaction set based on the simple mapping from *E. coli* to *PA* will lack coverage.

To increase both accuracy and coverage of the reference dataset, we constructed a positive reference dataset of *PA* PPIs mapped from large-scale PPIs of three closely related organisms: *C. jejuni, E. coli,* and *H. pylori*. Each mapped PPI was weighted according to the frequency of occurrence in source organisms, the confidence of the PPI in the source organism, and the evolutionary distance between the source organism and *PA*. The resulting positive reference dataset contains 3,629 interactions above a weight threshold, larger than possible datasets constructed from PPIs in any single organism [Table S1a]. The negative reference dataset contains 181,450 random interactions that include all 5,568 proteins in *PA*.

### Feature Collection, Compilation, and Ranking

We next collected various genomic data for each *PA* gene or its protein product from well-maintained sources. Genomic features were then compiled for each pair of *PA* genes or their protein products. Eight features with potential predictive power about protein physical interactions were considered in this study: co-**ess**entiality (ESS), co-**exp**ression (EXP), co-**fun**ctionality (FUN), co-**loc**alization (LOC), **d**omain-**d**omain **i**nteraction (DDI), co-**pat**hway involvement (PAT), **tr**ansmembrane **h**elices (TRH), and co-**op**e**r**on and co-gene cluster involvement (OPR).

A gene is considered essential or non-essential based upon survival of the organism under defined conditions. ESS captures the essentiality of a gene/protein pair. Because essential genes tend to encode hubs and interact with each other in the PPI network, protein pairs that are co-essential are more likely to interact physically [26], [31]. EXP measures whether two genes have similar expression patterns, as measured by the Pearson correlation coefficient of their mRNA expressions [26], [32]. Physically interacting proteins tend to have similar/same functions, and FUN captures whether a pair of proteins have at least one common function [26], [33]. Interacting proteins should have the same subcellular localization or domains that exist in the same locale, and LOC indicates whether two proteins are co-localized [25], [34]. Proteins physically interact through interactive domains. A pair of proteins that contain known interacting domains tend to interact with each other physically, which is captured by DDI [35]–[37]. Interacting proteins might have a better chance to be observed in the same pathway, and PAT denotes whether two proteins are involved in the same pathway [38], [39]. TRH indicates whether two proteins may both have transmembrane helices, which might also be an indication of their physical interaction [40]. Protein products of genes in the same operon are transcribed together, and these proteins are more likely to interact: OPR captures if a pair of genes are from the same operon or cluster [26], [41]. Among the eight features, seven of them except for EXP have nominal values. The feature value distribution for each of the features between positive and negative reference interactions can be found in **Table S2**.

To assess the relative predictive power of the features, we ranked all eight features using popular feature ranking methods and provided an average ranking for each feature [**Table 1**]. The chi-squared feature evaluation method evaluates each feature by measuring their chi-squared statistic with respect to the classes [42]. Gain ratio [43], information gain [44], and symmetric uncertainty [45] are commonly used entropy-based feature evaluation methods with different realization of feature importance with respect to the class labels. FUN, OPR, and PAT were ranked among the top three, their value distribution being different in positive against negative reference interactions intuitively [**Table S2**]. For example, for FUN, protein pairs with the same function(s) that interact (1,218) are almost as many as those that do not interact (1,330), while protein pairs with different functions have small chance to interact physically (2,411 interactions vs. 180,120 non-interactions); *p-value* <1e-5, one-sided Fisher’s exact test.

**Table 1:** Ranking of features to assess their relative predictive power.

Feature	Chi-squared FeatureEvaluation [42]	Gain Ratio [43]	Information Gain [44]	Symmetric Uncertainty [45]	Average Rank
FUN	1	2	1	1	1
OPR	2	1	3	2	2
PAT	3	3	4	3	3
LOC	5	5	2	5	4
DDI	6	4	6	4	5
ESS	4	6	5	6	6
EXP	7	7	7	7	7
TRH	8	8	8	8	8

### PPI Prediction

**Figure 1:**
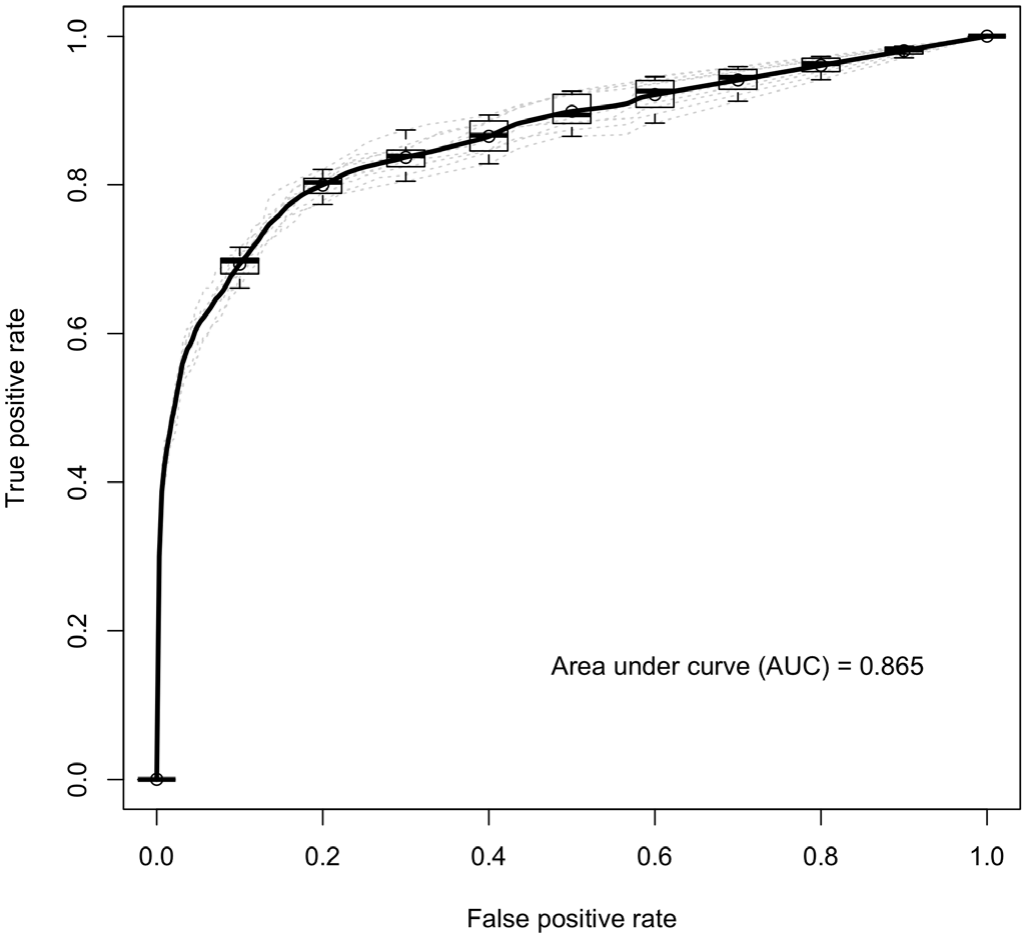
Receiver operator characteristic (ROC) curves of testing by 10-fold cross-validation. The ROC curve indicate the performance of 10-fold cross-validation by using eight features. The area under curve (AUC) is 0.865.

Based on the reference datasets and all eight features, we have trained and tested a random forest classifier that outperforms various other classification models including support vector machines, Bayesian networks, logistic regression, and artificial neural networks, in this study [**Table S3**]. 10-fold cross validation using all eight features yields an area under receiver operator characteristic curve (AUC) score of 0.865 [**Table 2** **;** **Figure 1** **; Figure S1**]. The excellent AUC score indicates both the effectiveness of the classification method and the high quality of the reference dataset. The precision and recall scores for the positive class are 0.659 and 0.414, respectively, with a false positive rate of 0.003. This implies strong performance, albeit with an influence by the dominance of negative interactions, or sparseness in PPI networks.

**Table 2:** Performance of the random forest classifier for the positive class in 10-fold cross-validation.

Features used for cross-validation	TP Rate	FP Rate	Precision	F-measure	AUC
FUN and OPR	0.118	<0.001	0.907	0.209	0.684
FUN, OPR, and PAT	0.267	0.002	0.717	0.389	0.705
Seven features, no TRH	0.279	0.003	0.642	0.389	0.855
All eight features	0.301	0.003	0.659	0.414	0.865

Following training and validation, we applied the classifier to label unknown protein pairs for physical interactions. The number of protein pairs to be predicted is remarkably large (15,313,449), consisting of all possible pairs between *PA* proteins except those in the reference dataset. The classifier predicted a total of 54,107 positive interactions, including 4,181 proteins with a probability higher than 0.5. Together with the positive reference set, the resulting *PA* interactome consists of 4,255 proteins and 57,736 interactions, covering the majority of the *PA* proteome (4,255 out of 5,568 *PA* protein ORFs, or 76.4%) [**Table S1b**]. Although the number of predicted PPIs is large, applying a threshold of high probability yields a smaller set of high-confidence predicted interactions. For example, with 0.9 as the confidence cutoff, a resulting high confidence set contains 15,777 interactions between 3,207 proteins [**Table S1c**]. Combining this high-confidence predicted dataset with the positive reference set results in a set of 19,406 interactions between 3,341 proteins.

### Validating Predicted PPIs

To verify the predicted PPIs, we performed validations based on three independent criteria. We first used identified experimental PPIs in *PA* that are not included in the positive reference dataset. We then analyzed the network structure and properties of the predicted network. Finally, we examined the position of known drug targets in the predicted network.

We extracted a set of 35 experimentally verified PPIs from MPIDB [11], 32 of which are not included in the positive reference set. Many of these PPIs are between protein products of neighboring genes. Of the 32 PPIs, 22 were predicted positive by the random forest classifier [**Table 3**]. In contrast, when using orthologs mapping from known PPIs of other bacteria organisms, none of the 32 PPIs can be predicted as positive [**Table 3**]. In bacteria such as *PA*, genes that are close to each other are often organized in operons, and the protein products of the genes from the same operon or neighboring genes tend to interact with each other. These interactions are identifiable by gene neighborhood-based methods, which will miss other PPIs between protein products of non-neighboring genes [41]. In contrast, our approach can also identify interactions between protein products of non-neighboring genes, for example *acpP2-spoT* and *qscR-rhlR*. This indicates better performance of our integrative approach compared with commonly used orthologs mapping. Combining the 10 verified but not predicted PPIs with predicted high-confidence and reference PPIs results in a high-confidence PPI network of 3,343 proteins and 19,416 interactions, henceforth referred to as the high-confidence PPI network. It is an ideal PPI network with which to carry out further validations and analysis.

**Table 3:** Validation of predicted PPIs by experimentally verified interactions.

Protein 1 ID	Protein 1 Symbol	Protein 2 ID	Protein 2 Symbol	In the positivereference set?	Confidence (if predicted positive)
PA0425	*mexA*	PA0426	*mexB*	Yes	
PA0425	*mexA*	PA0427	*oprM*		0.933
PA0426	*mexB*	PA0427	*oprM*		1
PA0763	*mucA*	PA0762	*algU*		1
PA0763	*mucA*	PA0764	*mucB*		0.967
PA0763	*mucA*	PA4446	*algW*		
PA0843	*plcR*	PA0844	*plcH*		1
PA1156	*nrdA*	PA1155	*nrdB*		1
PA1249	*aprA*	PA1250	*aprI*		
PA1454	*fleN*	PA1097	*fleQ*		
PA1665		PA0074	*ppkA*		
PA1665		PA0090	*clpV1*		
PA1706	*pcrV*	PA1705	*pcrG*		0.733
PA1707	*pcrH*	PA1708	*popB*		0.894
PA1709	*popD*	PA1707	*pcrH*		0.933
PA1709	*popD*	PA1708	*popB*		0.828
PA1710	*exsC*	PA1711	*exsE*		0.667
PA1714	*exsD*	PA1710	*exsC*		
PA1714	*exsD*	PA1713	*exsA*		
PA1869	*acpP2*	PA5338	*spoT*		0.551
PA1898	*qscR*	PA3477	*rhlR*		0.744
PA2494	*mexF*	PA2493	*mexE*		0.886
PA3008	*sulA*	PA4407	*ftsZ*		
PA3096	*xcpY*	PA3095	*xcpZ*		1
PA3101	*xcpT*	PA3097	*xcpX*		0.833
PA3101	*xcpT*	PA3098	*xcpW*		0.533
PA3101	*xcpT*	PA3099	*xcpV*		0.7
PA3101	*xcpT*	PA3100	*xcpU*		0.636
PA3101	*xcpT*	PA4525	*pilA*		
PA3104	*xcpP*	PA3105	*xcpQ*		0.547
PA3363	*amiR*	PA3374	*amiC*		0.967
PA4003	*pbpA*	PA4001	*mltB*		0.933
PA4407	*ftsZ*	PA5227	*zapA*		
PA5255	*algQ*	PA0576	*rpoD*	Yes	
PA5338	*spoT*	PA2966	*acpP1*	Yes	

The network structure and properties can be used to validate predicted PPIs [46]. The whole predicted network, the predicted network with only high-confidence edges, and the high-confidence PPI network all display scale-free topology with the degree exponent ranging from 1.34 to 1.69 [**Figure S2**]. Network statistics such as degree and clustering coefficient of the predicted networks are in accord with other PPI networks. For example, the high-confidence network contains 3,343 proteins and 19,416 interactions. Its average degree, clustering coefficient, and shortest path length are 11.62, 0.22, and 4.37, respectively. The high clustering coefficient and low shortest path length indicate desired high modularity and small world properties in the PPI network [46]. In addition, essential proteins are found more likely to be topologically important in the high confidence network. Out of 478 essential proteins in the network, 237 are hubs and 214 are bottlenecks, significantly higher than expected (p-values <1e-5 by Fisher’s exact tests; hubs and bottlenecks are defined as proteins that have the top 20% degree and betweenness centrality values in the network, respectively), consistent with the high correlation between essentiality and topological importance [47]. These results reinforce the validity of the network structure.

If the predicted interactome is valid, functionally important proteins such as drug target proteins should exhibit topological importance. Indeed, we found that known drug targets of *PA* tend to be hubs in the predicted network. We extracted 23 proteins that are approved drug targets related to *PA* infections from DrugBank [48], 20 of which exist in the high-confidence PPI network [**Table S4**]. Twelve of the 20 proteins are hubs in the high-confidence PPI network, significantly more than expected (p-value = 7.35e-5 by a one-sided Fisher’s exact test). In addition, six of the 23 drug target proteins that are essential are all hubs in the high-confidence PPI network, a finding consistent with the high correlation between essentiality and topological importance [47].

### Network-based Drug Target Identification and Prioritization

Based on the fact that drug targets are likely to be essential, hubs, and bottlenecks in the predicted high-confidence network (p-values are 3.0e-2, 1.4e-4, and 9.4e-4, respectively, by Fisher’s exact tests), we designed a simple method to rank and prioritize potential drug targets by incorporating both topological importance and essentiality. In additional to being essential, the higher the degree and betweenness values a protein has, the higher the rank it receives (see Methods). The resulting list yields a total of 276 proteins that are prioritized for further filtering [**Table S5a**]. Filters were then applied to the list based on a set of properties that an ideal drug target should possess, including: (a) a relatively well elucidated function, (b) no close homologs in humans to reduce toxicity, and (c) location in the cytoplasmic membrane, periplasm, or outer membrane to be easily accessible by drugs. After the filtering, 28 of 276 proteins were selected to serve as potential drug targets that may merit further investigation [**Table S5b**].

### Modular Analysis Reveals Essential Modules and Potential Modular Drug Targets

Protein networks consist of modular subnetwork structures that perform specific functions [49], [50]. To identify functional modules from the *PA* PPI network, we applied a widely used molecular complex detection method, MCODE, on the high-confidence PPI network [51]. This method selects densely connected subnetwork regions and ignores the rest of the network. It yielded 113 modules from the high-confidence network, including 1,154 proteins and 2,909 interactions [**Table S6a**]. Interestingly, although identified modules cover only about one third of all proteins in the network, the majority of essential genes in the high-confidence network (274 of 328, or 83.5%) remain in the modules, reflecting their functional essentiality by topological importance. In addition, 220 of 274 (or 80.3%) essential proteins were found in 31 of 113 (or 27.4%) modules that contain at least 25% essential proteins, suggesting that these essential proteins tend to interact with other essential proteins and form “essential modules” that perform designated functions. Notably, nine of 20 known drug targets in the network (PA0004 gyrB, PA3168 gyrA, PA3482 metG, PA3834 valS, PA3987 leuS, PA4238 rpoA, PA4268 rpsL, PA4269 rpoC, and PA4967 parE) were involved in identified modules. Eight of them, PA3987 leuS being the sole exception, were in “essential modules”, reflecting the importance of these essential modules.

We further identified the over-represented functions in identified modules. Despite the highly insufficient functional annotations for *Pseudomonas* proteins (only 1,519 or 27.3% of all 5,568 *PA* protein ORFs are annotated with Gene Ontology terms [33] to date), 32 out of 113 modules were found enriched in at least one function based on the Gene Ontology annotations (p-value <1e-3 by a Fisher’s exact test) [33] [**Table S6b**]. In addition, over-represented functions were found in all six modules that contain the nine known drug targets. Several enriched functions that are common in at least two modules include *cellular amino acid metabolic process* (GO:0006520), *cellular protein metabolic process* (GO:0044267), *cofactor biosynthetic process* (GO:0051188), *nucleotide metabolic process* (GO:0009117), and *RNA metabolic process* (GO:0016070), all of which are essential functions for the survival of the organism.

**Figure 2:**
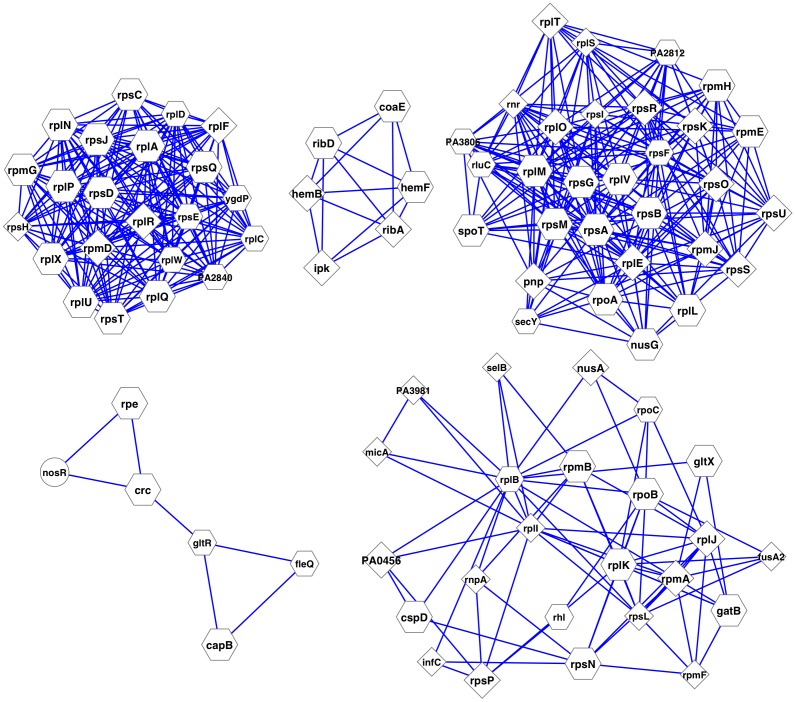
The top five essential modules that may contain multiple potential drug targets. In the figure, a diamond node represents a hub protein, and a hexagon node represents a hub and bottleneck protein in the high-confidence network. Larger nodes indicate essential proteins, and smaller ones are non-essential. The majority of module members are hubs and/or bottlenecks in the network, reflecting their essentiality. Figures 2, 3, and 4 were drawn using Cytoscape [72].

Based on the topological and functional importance of essential modules, we have devised a method to rank and prioritize these essential modules that may contain multiple proteins to be targeted simultaneously by drug molecules. An integrative score is associated with each essential module. The score is calculated by combining measures based on the percentage of essential proteins, the percentage of topologically important proteins, and the existence of over-represented functions [**Table S6c**] (see Methods). The higher the score, the more topological and functional importance a module exhibits. Protein members in highly important modules may be selected and targeted simultaneously. The top five essential modules that might contain multiple potential drug targets are illustrated in **Figure 2**. For example, one of the top ranked modules contains six protein members (PA4047 ribA, PA0024 hemF, PA4529 coaE, PA4056 ribD, PA4669 ipk, and PA5243 hemB), all of which are both essential and hubs in the high-confidence network and three of which (PA0024 hemF, PA4529 coaE, and PA4056 ribD) are bottlenecks in the network. These six essential proteins are enriched in *cofactor biosynthetic process* (GO:0051188; p-value = 8.9e-9) and *riboflavin biosynthetic process* (GO:0009231; p-value = 3.6e-5). Four of the six essential proteins (except PA4529 coaE or PA5243 hemB) that do not have human orthologs might be targeted simultaneously by drug molecules.

### A Map of Human-Pseudomonas Interactions Supports Identified Potential Drug Targets

**Figure 3:**
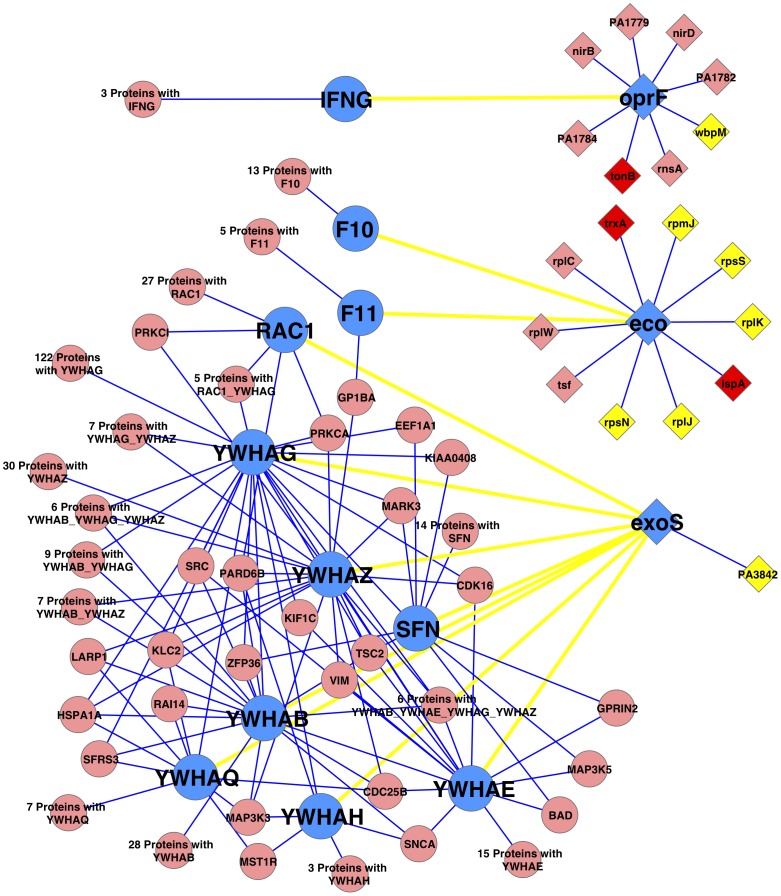
A map of human-*P. aeruginosa* protein interactions. In the figure, a round node represents a human protein, and a diamond node represents a PAO1 protein. Blue nodes are proteins involved in human-Pseudomonas protein interactions that represented by yellow edges, based on the data from the pathogen interaction gateway [52]. Blue edges denote corresponding protein-protein interactions in a human interactome and the high-confidence PA PPI network. Yellow and red PAO1 proteins are essential proteins, and red ones are predicted to be potential drug targets by this study. The full lists of proteins for abbreviated nodes, e.g., ‘30 Proteins with YWHAZ’, can be found in Table S7c.

The infectious process of bacterial pathogenesis often involves interaction of bacterial and host proteins. Thus, a map of human-*PA* protein interactions will arguably help elucidate the disease mechanisms of CF and COPD triggered by *PA* infection. We extracted and processed 12 human-*PA* protein interactions between 11 human proteins and three *PA* proteins from pathogen interaction gateway (PIG) [52], either from direct human-*PA* interactions or by human-*E. coli* interactions and ortholog mapping between *E. coli* and *PA* proteins [**Table S7a**]. Together with the interactions from a human PPI network from human protein reference database (HPRD) [53] and those from the high-confidence PPI network from this study that contain proteins involved in human-*PA* protein interactions, a map of human-*PA* interactions was constructed [**Figure 3**] [**Table S7b and S7c**]. Top over-represented GO annotations by human proteins in the network are *cytosol* (GO:0005829) and *cytoplasm* (GO:0005737) of cellular components, *protein binding* (GO:0005515), *nucleotide binding* (GO:0000166), *protein serine/threonine kinase activity* (GO:0004674), and *ATP binding* (GO:0005524) of molecular functions, and *nerve growth factor receptor signaling pathway* (GO:0048011), *blood coagulation* (GO:0007596), *intracellular signaling pathway* (GO:0023034), and *platelet activation* (GO:0030168) of biological processes (p-value <1e-12) [Table S8]. *Cellular protein metabolic process* (GO:0044267) is enriched among *PA* proteins (p-value = 7.9e-6). Surprisingly, 10 of 22 *PA* proteins in the map are essential, and three proteins (TonB, thioredoxin TrxA, and lipoprotein signal peptidase LspA) were predicted as potential drug targets by this study [**Table S5b**], with both numbers being significantly higher than random expectation (p-values are 1.6e-5 and 5.2e-4, respectively). This intensified relevance of the proteins in the map supports the validity of them as potential drug targets, and further investigations are required to experimentally verify this hypothesis.

### Case Studies: Anti-sigma Factor MucA, Negative Periplasmic Alginate Regulator MucB, and the Quorum Sensing Transcriptional Regulator RhlR

**Figure 4:**
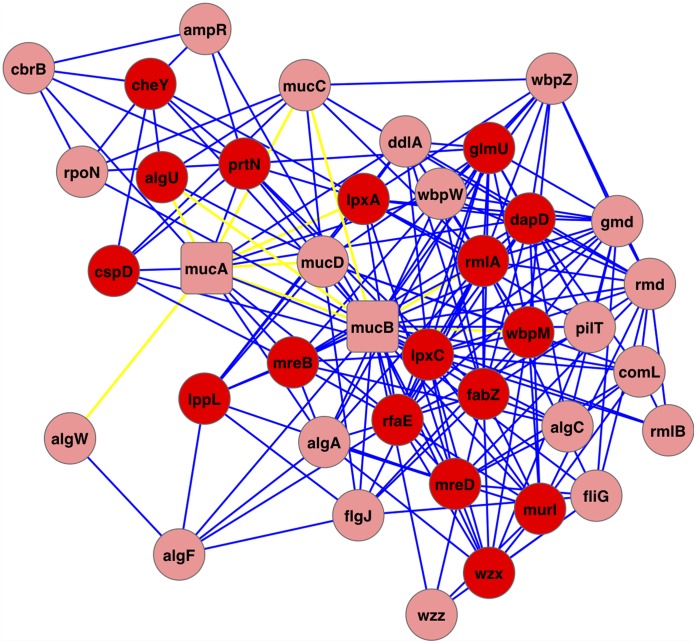
A level-1 interaction map for MucA and MucB. Each node is a protein and each edge is a predicted PPI from the high-confidence network (except the interaction MucA-AlgW, which comes from experimental PPI data). A total of 39 proteins and 199 interactions were captured by the level-1 PPI network for MucA and MucB. 17 Red nodes are essential proteins. Yellow edges indicate high confidence interactions included in the high-confidence network.

As reviewed by Hassett et al. [54], the cytoplasmic membrane bound anti-sigma factor MucA and the transcriptional regulator RhlR are promising *PA* drug targets of CF airway disease. There is burgeoning evidence that during chronic CF airway disease, the airway mucus has either significantly reduced oxygen tension or is, in fact, anaerobic. Mutant strains lacking MucA or RhlR are either exquisitely sensitive to slightly acidified sodium nitrite (NaNO_2_) [29] or commit a metabolic suicide by overproduction of NO during anaerobic respiration [30], respectively. Using our integrative approach, 15 and 29 interacting partners are predicted for anti-sigma factor MucA and related negative periplasmic alginate regulator MucB, respectively, with a confidence cutoff of 0.5. Six common interacting partners are shared by MucA and MucB, including AlgT(U), MreB, MucC, MucD, PrtN, and RfaE, respectively, and *mucABCD* are in the same module with high connectivity [**Table S6a**]. A level-1 PPI network of 39 proteins and 199 interactions is constructed among MucA, MucB, and their interacting partners, capturing all predicted interactions among these proteins [**Figure 4**]. Essential proteins are significantly enriched in this rather densely connected subnetwork (17 of 39 proteins are essential; p-value = 6.9e-6). The importance of this subnetwork to the survival of the pathogen highlights the potential of its protein members as druggable targets. Top over-represented functions by MucA, MucB, and their interacting partners include *lipopolysaccharide biosynthetic process* (GO:0009103) and *Gram-negative-bacterium-type cell wall* (GO:0009276) (p-value <1e-5) [Table S9a].

The transcriptional regulator RhlR is a regulatory hub that interacts with 60 partners in the predicted protein interaction network, many of which are transcription factors or two-component regulators [**Figure S3**]. The components of the RhlR-focused subnetwork are well reflected by enriched functions of the protein members, including *transcription regulator activity* (GO:0030528), *two-component response regulator activity* (GO:0000156), *regulation of transcription, DNA-dependent* (GO:0006355), *two-component signal transduction system* (phosphorelay) (GO:0000160), and *response to stimulus* (GO:0050896) (p-value <1e-5)[Table S9b].

### Web Server

**Figure 5:**
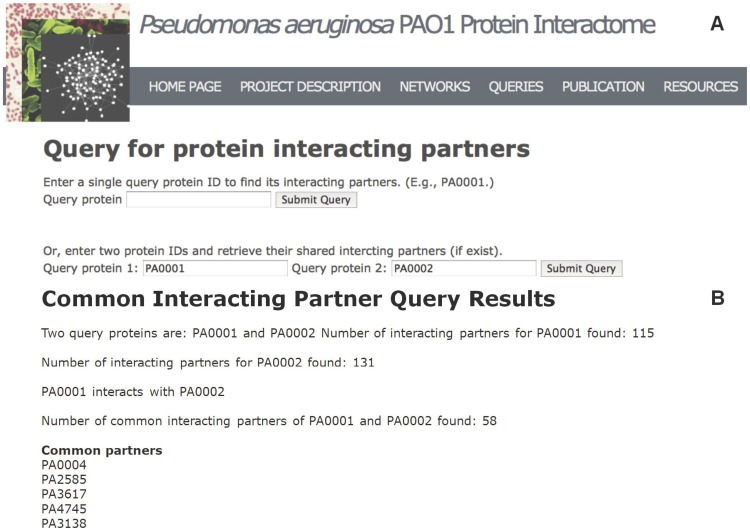
A screen shot of the Web server of the *PA* PPI interactome. (A) To query interacting partners based on our predicted PA PPI interactome, users can either query PPIs of a single *PA* protein (e.g., PA0001) or query common interacting partners of two different *PA* proteins (e.g., PA0001 and PA0002). (B) The results of querying common interacting partners of two *PA* proteins include the number of interacting partners for each protein, whether the two query proteins interact, and all common partners (only five were shown in the figure).

We have developed a Web server to store and display the predicted PPIs [**Figure 5**]. The Web server can be accessed at http://research.cchmc.org/PPIdatabase/. We used techniques including HTML, XML, CSS, and PHP to construct the Web page and MySQL to build the database for query. Our Web server provides query and download services of the identified *PA* PPI interactome. The interface is simple and straightforward. Specifically, users can perform two types of queries: query the interacting partners of particular PA proteins and query common interacting partners of two selected *PA* proteins. Three networks are available for download: the whole *PA* PPI interactome (combining all predicted and reference PPIs), the high-confidence PPI network as used in our analyses, and the PPIs involved in host-pathogen interactions.

## Discussion

In this study, we employed a supervised learning approach to generate a protein interactome map for the important pathogen *P. aeruginosa* PAO1 (*PA*), an organism that is particularly problematic in chronic airway infections of CF and COPD patients. The main advantage of machine learning-based approaches to mapping PPIs based on sequence similarity lies in the high coverage of the prediction, because the predictions are not restricted to proteins with orthologs in other organisms. Indeed, the interactome map generated from our study covers the majority of *PA* proteins (4,255 out of 5,568, or 76.4%) while recent studies on predicting interactome maps of *Pseudomonas putida* KT2440 and *Leishmania major* reached only 60.8% and 43.6% of all *P. putida* KT2440 and *L. major* proteins, respectively [55], [56].

A successful prediction by a supervised learning approach relies on the quality of the reference set, the relevance of selected features to the classification, and the specific machine learning algorithm. For a non-model organism such as *PA*, the reference set assembly is challenging because few experimentally verified interactions exist. To construct a reference set of good coverage and accuracy, instead of using PPIs from a single model organism, we used a novel approach, combining multiple large-scale PPI datasets from closely related bacteria species (*C. jejuni*, *E. coli*, and *H. pylori*). Potential *PA* PPIs were mapped from these datasets via ortholog mapping, and each mapped *PA* interaction was associated with a confidence score. The resulting reference set covers more PPIs than one that is mapped from any single PPI dataset of a model organism, and its quality is retained by selecting only high-confidence PPIs based on a ranked list of all PPIs. This approach of constructing reference sets may be applied for the interactome network predictions in other non-model organisms. For genomic features used for classification in this study, we selected those that have been proven useful in PPI predictions [25], [26], [31], [32], [34], [37], [39]–[41]. The random forest classifier was chosen because of its superior performance over many other algorithms and methods in the training and testing, including decision trees, several regression models, support vector machines, and Bayesian network-based methods [Table S3].

Recent developments in network pharmacology suggests that the selection of potential drug target proteins may be based on their relationships with other members in a molecular network in addition to their individual biochemical properties [9], [55], [57]. In our study, in addition to identifying and prioritizing potential drug targets based on network topology and protein essentiality, we identified groups of multiple potential target proteins from extracted modules with essential functions. These proteins may correspond to members of the same pathway or the same complex and together provide alternative mechanisms to perform the same essential function(s) for the overall survival of *PA*. Drug resistance, for example, might thus be relieved by disrupting the functioning of essential modules via simultaneously targeting multiple of their essential members. In this study, we employed a network-based systematic approach and identified both individual and inter-related putative drug target proteins. Hypotheses concerning the effectiveness of these drug targets will be tested via future experimental verifications.

## Materials and Methods

### Data Sources

Protein sequences of all 5,568 *P. aeruginosa* PAO1 open reading frame proteins were downloaded from the *Pseudomonas* Genome Database (http://www.pseudomonas.com/). Gene expression data of wild type PAO1 under normal condition were collected from GEO and ArrayExpress (http://www.ncbi.nlm.nih.gov/geo/) [58]. Functional annotations for PAO1 genes were extracted from GO [33]. Pathway information of strain PAO1 was retrieved from KEGG [59]. A list of essential genes of PAO1 was downloaded from Database of Essential Genes (DEG) [60].

### Construction of the Reference Datasets

We collected large-scale experimentally determined PPI data of three bacteria species (*C. jejuni*, *E. coli*, and *H. pylori*) that are closely related to *PA* from DIP, BIND, and literature [61]–[63] (http://bond.unleashedinformatics.com/) [Table S1d]. We then associated a weight with each PPI according to the evolutionary distance between the particular bacterium and *PA*, as calculated by PHYLIP [64]. If a PPI is in a core set with high-confidence, 0.2 is added to its weight. PPIs in each source organism were then mapped to *PA* based on strict orthologs by a reciprocal best hit approach between the organism and *PA* [65]. The weight of each mapped PPI is summed from the weights of corresponding PPIs in the source organisms, and the interactions that exist in multiple organisms receive more weight. 3,629 mapped PPIs between 1,215 proteins that have weight higher than 0.8 are considered positive reference interactions in *PA*. Negative reference interactions were 181,450 randomly selected between protein pairs that do not overlap with positive reference interactions.

### Feature Collection and Compilation

#### (1) Co-expression data (EXP)

Gene expression data of 18 wide type *PA* PAO1 samples under normal conditions were extracted from public data sources GEO and ArrayExpress (http://www.ncbi.nlm.nih.gov/geo/) [58]. Pearson correlation coefficients were then calculated for the expression levels of every pair of genes in 18 samples. We used numerical values of these coefficients as a feature vector.

#### (2) Co-functionality (FUN)

Functional annotations of *PA* PAO1 proteins were extracted from Gene Ontology database [33]. A total of 4,227 non-redundant functional annotations exist for 1,520 *PA* proteins, covering less than one third of all *PA* proteins. Due to the relatively small coverage of functional annotations, we define the co-functionality value for any pair of genes as a binary value that is either true (the two genes have at least one common function) or false (no common function is shared by a pair of genes).

#### (3) Co-essentiality (ESS)

Gene essentiality data were retrieved from DEG [60]. Each gene of *PA* PAO1 is either essential (678 genes) or non-essential (4,890 genes). Based on gene essentiality, every pair of genes has a categorical value for co-essentiality: both being essential, both being non-essential, one being essential and the other non-essential.

#### (4) Co-pathway involvement (PAT)

The pathway information of *PA* PAO1 genes were retrieved from KEGG [59]. A protein is either involved in a pathway or not. The co-pathway involvement score for a pair of proteins has a binary value indicating whether the two proteins are in the same pathway.

#### (5) Co-localization (LOC)

Five different localization motifs exist for PAO1 proteins: cytoplasmic membrane, extracellular, cytoplasm, periplasm, and outer membrane. The subcellular localization data for each PAO1 protein is predicted by Proteome Analyst Specialized Subcellular Localization (PASSL) Server v2.5 (http://www.cs.ualberta.ca/~bioinfo/PA/Sub/). A categorical co-localization value is then compiled for each pair of proteins. A pair of proteins can either have the same or different predicted subcellular localization, or none prediction can be made for at least one of the proteins.

#### (6) Domain-domain interaction (DDI)

Two proteins are likely to interact with each other if they contain interacting domains. The domain-domain interaction data are predicted from DOMINE [66]. And the domain information of each PAO1 protein is retrieved from Pfam database [67]. The feature value for DDI is a binary value, indicating either or not predicted domain-domain interaction exists between a protein pair.

#### (7) Transmembrane helices (TRH)

The transmembrane helix is a domain structure type useful to describe the gene sequences. Combining this feature may help us to find the essential genes cluster in features space. The potential helices for PAO1 proteins are predicted by the TMHMM Server v. 2.0 (http://www.cbs.dtu.dk/services/TMHMM/). The TRH feature is a binary feature that describes whether both proteins have predicted transmembrane helices for each pair of proteins.

#### (8) Co-operon involvement and gene clusters (OPR)

The operon information for PAO1 genes were downloaded from database of prokaryotic operons (DOOR) [68]. Gene cluster information is from the *PA* PAO1 genome database (http://www.pseudomonas.com/). The OPR feature value is a binary value describes whether or not two proteins are from the same operon or cluster.

### Random Forest Classifier

We used a random forest classifier as the learning method [69]. A random forest method is an ensemble classifier of many decision trees. The output class of the method is the class that occurs most frequently by different decision trees. We used the Weka implementation of the random forest classifier, and selected the parameters as four features for each decision tree, 30 decision trees and 10 seeds [70].

### Ranking Potential Drug Target Proteins

Essential proteins in the high-confidence network were ranked according to their degree values and betweenness values. In addition, an overall rank score is associated with each essential protein combining its ranks in degree and betweenness values. Specifically, a score is associated with each essential protein *P* as
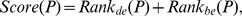
where *Rank_de_*(*P*) and *Rank_be_*(*P*) are the ranks of degree and betweenness values, respectively, for protein *P*. The score indicates the overall rank of the importance of a protein: The lower the score is, the higher final rank the protein receives. For example, the overall rank of a protein which is the top number 5 in its degree value and the top number 4 with a *Score* of 9 in its betweenness value is high than one which is the top number 4 in its degree value and the top number 6 in its betweenness value with a *Score* of 10.

### Ranking Potential Modular Drug Targets

Essential modules extracted from the high-confidence network were ranked based on an integrative score combining the measure of the percentage of essential protein members, the percentage of hubs and bottlenecks, and the existence of over-represented function(s). Specifically, a score is associated with each module *M* as
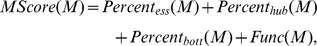
where *Percent_ess_*(*M*), *Percent_hub_*(*M*), and *Percent_bott_*(*M*) are the percentages of essential proteins, hubs, and bottlenecks in module *M*, respectively, and *Func*(*M*) is 1 if module *M* has at least one enriched function or 0 otherwise.

### Filtering Potential Drug Target Proteins

We applied three independent filters after obtaining 237 essential proteins in *PA* that are topologically important in the predicted high-confidence network. First, two proteins were filtered out due to no known functional annotation from GO. Second, 43 more proteins were filtered out because they have orthologous proteins in humans determined by a reciprocal best hit method based on Blastp [71] [65]. Finally, 167 remaining proteins that do not have subcellular localization in membrane or periplasm were filtered out, yielding a final list of 25 proteins that are likely to be potential drug targets. The subcellular localization data were obtained from prediction by PASSL Server v2.5 (http://www.cs.ualberta.ca/~bioinfo/PA/Sub/).

### Identifying Host-pathogen Interactions between Human and PA Proteins

We used the pathogen interaction gateway database to extracted human-pseudomonas protein interactions [52]. Host-pathogen protein interactions between human and *PA* (nine human proteins, two *PA* proteins, and nine interactions) and those between human and *E. coli* (18 human proteins, four *E. coli* proteins, and 19 interactions) were extracted. One of the four *E. coli* proteins can be mapped to one *PA* protein based on homology mapping by a reciprocal best hit method [65], resulting in two additional interactions between human and *PA* proteins (two human proteins and one *PA* protein).

## Supporting Information

Figure S1 **ROC curves of testing the random forest classifier.** ROC curves of testing the random forest classifier by 10-fold cross-validations using all eight features and using a subset of the eight features.
(DOC)

Figure S2 **Scale-free topology for predicted networks.** All four networks (the whole predicted network, the high-confidence predicted network, and these two networks with the positive reference set) have power-law degree distributions with the corresponding degree exponent ranging from 1.34 to 1.69.
(DOC)

Figure S3 **Interacting partners of transcription factor rhlR.** In the figure, each node is a protein, and each edge is an interaction from the predicted PA network. Yellow edges indicate high-confidence interactions based on our prediction, and red proteins denote essential proteins.
(DOC)

Table S1 **A summary of all PPI data used in training/testing and PPIs predicted.** PPIs in the positive reference dataset are listed in Table S1A. Table S1B and S1C show all predicted PPIs and the high confidence set, respectively. Table S1D lists the PPIs from the source organisms that were used to construct the reference dataset.
(XLSX)

Table S2 **Feature values of the eight features used.** Values of seven nominal features and one numerical feature are shown along with the class labels.
(XLSX)

Table S3 **Performance of different classification methods.** Performance of a variety of classification algorithms and methods is shown. Two different negative reference datasets of different sizes were used.
(XLSX)

Table S4 **Essentiality, hub status, and module membership of known drug targets.** The essentiality, existence in the networks and modules, and the hub status of 23 known drug targets are listed along with p-values by Fisher’s exact tests.
(XLSX)

Table S5 **Topologically important essential proteins and predicted drug targets**. Detailed network statistics, ranks, and other properties are listed for all essential proteins in the network (Table S5A) and the 28 potential drug targets (Table S5B).
(XLSX)

Table S6 **Identified modules, over-represented functions of their protein members, and potential modular drug targets.** All identified modules are listed in Table S6A. Functional enrichment of module members is shown in Table S6B. And essential modules are ranked in Table S6C.
(XLSX)

Table S7 **A summary of human-** ***PA*** **PPIs.** 12 human-*PA* PPIs are listed in Table S7A, and interacting partners of proteins involved in human-*PA* PPIs from *PA* and human are listed in Table S7B and S7C, respectively.
(XLSX)

Table S8 **Functional enrichment by human proteins involved in human-** ***PA*** **PPIs.** The most significantly enriched functions of the three GO categories are high-lighted.
(XLSX)

Table S9 **Over-represented functions by** ***PA*** **proteins involved in mucA, mucB-related PPIs and rhlR-related PPIs.** The top significantly enriched GO functional annotations are high-lighted.
(XLSX)
