# Supplementary material for: Prediction and Analysis of the Protein Interactome in Pseudomonas aeruginosa to Enable Network-Based Drug Target Selection
Source: PLoS One. 2012 Jul 24;7(7):e41202. doi: 10.1371/journal.pone.0041202 (PMC3404098; doi:10.1371/journal.pone.0041202)
Supplement: Figure S1 — ROC curves of testing the random forest classifier. ROC curves of testing the random forest classifier by 10-fold cross-validations using all eight features and using a subset of the eight features. (DOC) [file pone.0041202.s001.doc]

**Fig. S1. ROC curves for 10-fold cross-validation results**

ROC curves of testing the random forest classifier by 10-fold cross-validations using all eight features and using a subset of the eight features. The ROC curves were plotted by ROCR [1].

1. Sing T., Sander O., Beerenwinkel N. and Lengauer T. (2005) ROCR: visualizing classifier performance in R. Bioinformatics, 21 (20), 3940-3941.
