# Supplementary material for: Prediction and Analysis of the Protein Interactome in Pseudomonas aeruginosa to Enable Network-Based Drug Target Selection
Source: PLoS One. 2012 Jul 24;7(7):e41202. doi: 10.1371/journal.pone.0041202 (PMC3404098; doi:10.1371/journal.pone.0041202)
Supplement: Figure S2 — Scale-free topology for predicted networks. All four networks (the whole predicted network, the high-confidence predicted network, and these two networks with the positive reference set) have power-law degree distributions with the corresponding degree exponent ranging from 1.34 to 1.69. (DOC) [file pone.0041202.s002.doc]

**Figure S2. Scale-free topology for predicted networks**

All four networks (the whole predicted network, the high-confidence predicted network, and these two networks with the gold-standard positive set) have power-law degree distributions with the corresponding degree exponent ranging from 1.34 to 1.69. These figures are drawn by VisANT [1].

The whole predicted network


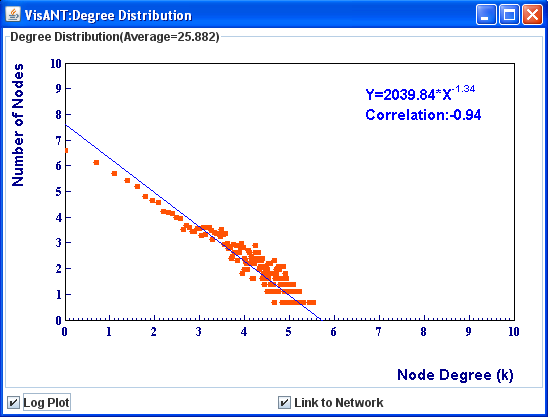


The whole predicted network combined with the gold-standard positive set


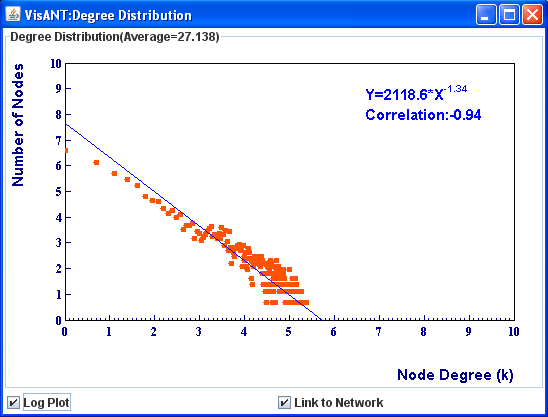


The predicted network with only high-confidence edges


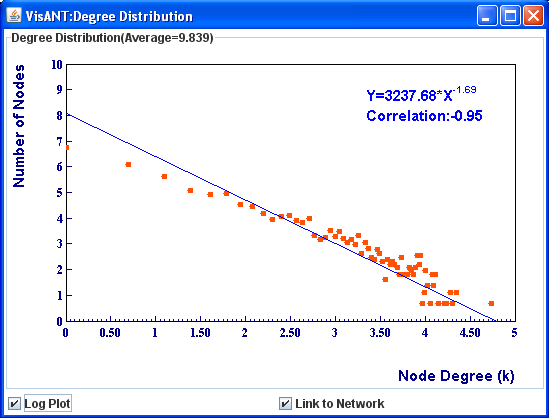


The predicted network with high-confidence edges and the gold-standard positive set


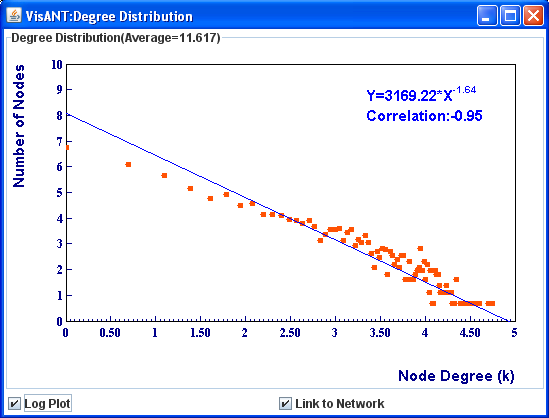


The high-confidence network (combining predicted high-confidence interactions, gold-standard positive interactions, and experimentally verified interactions)


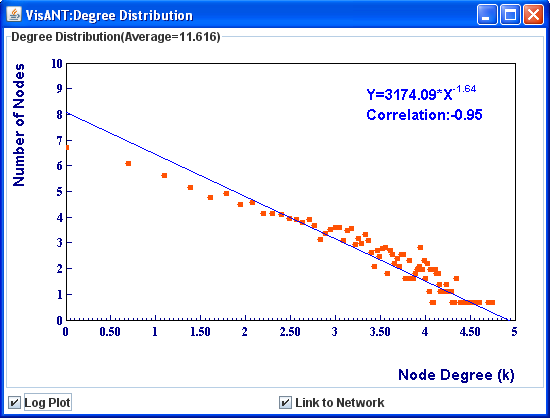


1. Hu, Z., E.S. Snitkin, and C. DeLisi, *VisANT: an integrative framework for networks in systems biology.* Brief Bioinform, 2008. **9**(4): p. 317-25.
