# Supplementary material for: Prediction and Analysis of the Protein Interactome in Pseudomonas aeruginosa to Enable Network-Based Drug Target Selection
Source: PLoS One. 2012 Jul 24;7(7):e41202. doi: 10.1371/journal.pone.0041202 (PMC3404098; doi:10.1371/journal.pone.0041202)
Supplement: Figure S3 — Interacting partners of transcription factor rhlR. In the figure, each node is a protein, and each edge is an interaction from the predicted PA network. Yellow edges indicate high-confidence interactions based on our prediction, and red proteins denote essential proteins. (DOC) [file pone.0041202.s003.doc]

**Figure S3. Interacting partners of transcription factor rhlR.**


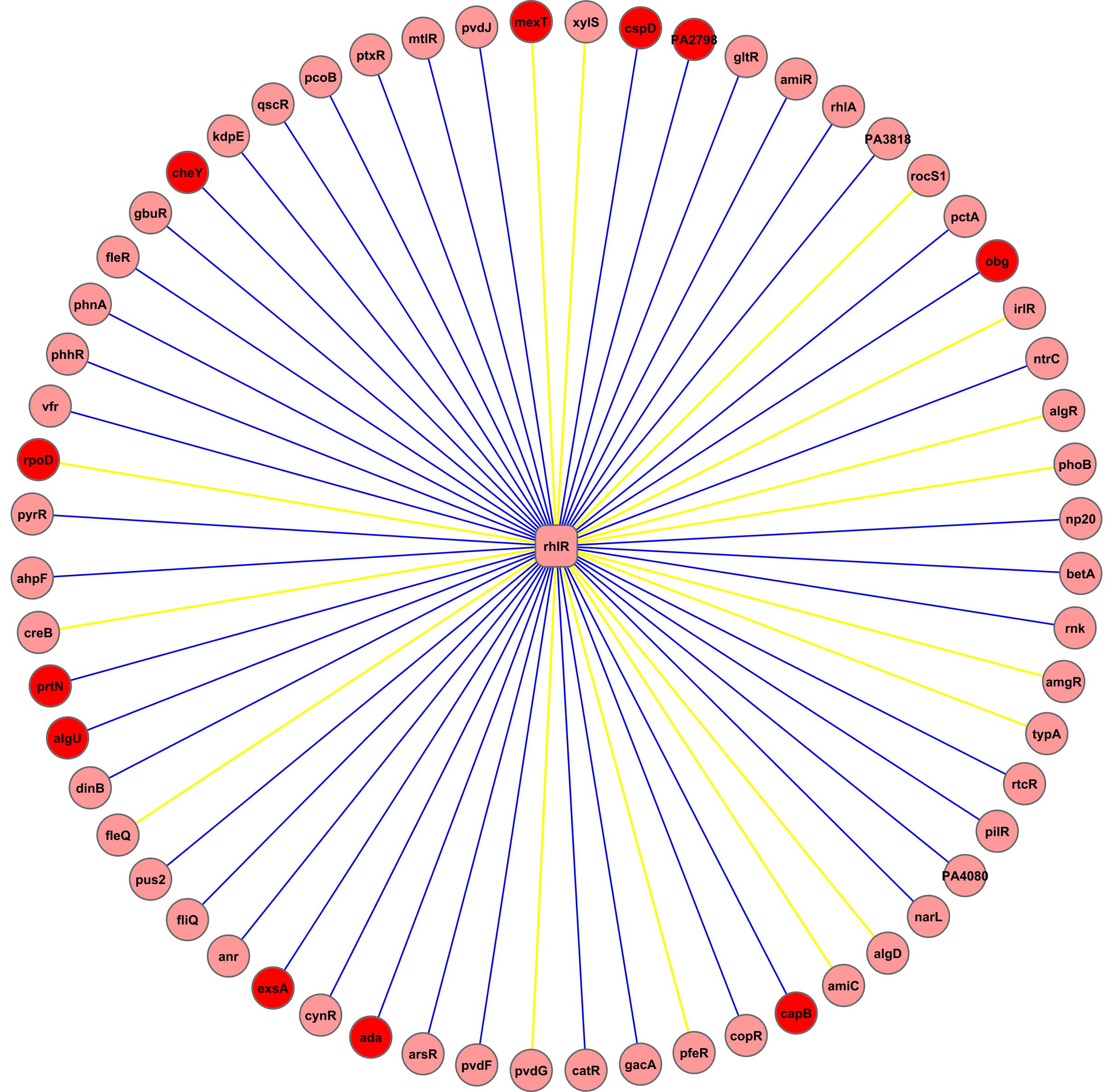


**Figure S3. Interacting partners of transcription factor rhlR.** In the figure, each node is a protein, and each edge is an interaction from the predicted PA network. Yellow edges indicate high-confidence interactions based on our prediction, and red proteins denote essential proteins. This figure was drawn by Cytoscape [1].

1. Shannon, P., et al., *Cytoscape: a software environment for integrated models of biomolecular interaction networks.* Genome Res, 2003. **13**(11): p. 2498-504.
